# A novel anti-LAG-3/TIGIT bispecific antibody exhibits potent anti-tumor efficacy in mouse models as monotherapy or in combination with PD-1 antibody

**DOI:** 10.1038/s41598-024-61477-6

**Published:** 2024-05-09

**Authors:** Tongcheng Dai, Hao Sun, Tyler Liban, Ildefonso Vicente-Suarez, Bin Zhang, Yongping Song, Zhongxing Jiang, Jifeng Yu, Jackie Sheng, Binhua Lv

**Affiliations:** 1 Suzhou Zelgen Biopharmaceuticals Co., Ltd, Kunshan, China; 2 The First Affiliated Hospital of Zhengzhou University, Zhengzhou, Henan China; 3 Gensun Biopharma Inc., Thousand Oaks, CA USA

**Keywords:** Lymphocyte-activation gene 3 (LAG-3), T cell immunoreceptor with immunoglobulin and ITIM domain (TIGIT), Bispecific monoclonal antibody, Immune checkpoint inhibitor, Cancer immunotherapy, Preclinical research, Drug development

## Abstract

We report the generation of a novel anti-LAG-3/TIGIT bispecific IgG4 antibody, ZGGS15, and evaluated its anti-tumor efficacy in mouse models as monotherapy or in combination with a PD-1 antibody. ZGGS15 exhibited strong affinities for human LAG-3 and TIGIT, with KDs of 3.05 nM and 2.65 nM, respectively. ZGGS15 has EC50s of 0.69 nM and 1.87 nM for binding to human LAG-3 and TIGIT on CHO-K1 cells, respectively. ZGGS15 competitively inhibited the binding of LAG-3 to MHC-II (IC50 = 0.77 nM) and the binding of TIGIT to CD155 (IC50 = 0.24 nM). ZGGS15 does not induce ADCC, CDC, or obvious cytokine production. In vivo results showed that ZGGS15 had better anti-tumor inhibition than single anti-LAG-3 or anti-TIGIT agents and demonstrated a synergistic effect when combined with nivolumab, with a significantly higher tumor growth inhibition of 95.80% (*p* = 0.001). The tumor volume inhibition rate for ZGGS15 at 2 mg/kg was 69.70%, and for ZGGS15 at 5 mg/kg plus nivolumab at 1 mg/kg, it was 94.03% (*p* < 0.001). Our data reveal that ZGGS15 exhibits potent anti-tumor efficacy without eliciting ADCC or CDC or causing cytokine production, therefore having a safe profile.

## Introduction

Despite the fact that targeted immunotherapy, such as anti-programmed death receptor-1/programmed death protein ligand-1 (PD-1/L1) monoclonal antibody (mAb) treatment, has improved efficacy and durability in multiple tumor types, the inhibition of a single immune checkpoint may not be enough to overcome immune suppression^1,2^. Tumors can evade the immune system through various biological mechanisms, rendering many patients nonresponsive to single agent therapies^3,4^. In Melanoma, more than 40% of patients are unresponsive to existing first-line nivolumab (PD-1 mAb) plus ipilimumab (cytotoxic T-lymphocyte-associated antigen 4 (CTLA-4) mAb) therapies^5^, with a growing unmet need for novel regimens to overcome the targeted immunotherapy resistance^6^.

Lymphocyte-activation gene 3 (LAG-3), as a type I transmembrane in the immunoglobulin (Ig) superfamily, is mainly expressed in activated T cells, NK cells, B cells, plasma cells, and dendritic cells (DCs). The expression of LAG-3 is negatively correlated with the immune regulatory function of specific T cells, and inhibiting LAG-3 function can enhance the anti-tumor effect of specific CD8^+^ T cells^7^. LAG-3 is also highly expressed on tumor-infiltrating lymphocytes (TILs) in a variety of cancers^8^ and has been considered an emerging immune checkpoint in the tumor microenvironment with implications for cancer immunotherapy^9^. Importantly, combination treatment of anti–LAG-3 and anti–PD-1 showed a synergistic effect and was superior to the efficacy of single agent blockades^7^.

One important inhibitory receptor of T cells and NK cells is the T cell immunoreceptor with immunoglobulin and ITIM domain (TIGIT). By interacting with Poliovirus receptor (PVR) and Poliovirus receptor-related 2 (PVRL2), TIGIT, which is expressed on human NK cells, can reduce the cytotoxicity of NK cells. TIGIT is also highly expressed on the surface of tumor-infiltrating T cells. TIGIT blockade increased Fas-L expression and IFN-γ production via the NF-κB signaling pathway, and blocking TIGIT/CD155 signaling enhanced the antitumor effect of expanded NK cells against castration-resistant prostate cancer^10^. TIGIT blockade enhanced PD-1 blockade-mediated cytokine production by CD8^+^ TILs in bladder cancer patients^11^. In a preclinical study, dual blockade of TIGIT and PD-1 can specifically enhance the anti-tumor effect of CD8^+^ T cells with synergistic inhibition efficacy, with the majority of the treated animals achieving a complete response (CR)^12^. Like LAG-3 and PD-1, TIGIT and PD-L1 synergistic blockades are superior to single blockades^13^.

Although anti-PD-1 combined with anti-TIGIT or anti-LAG-3 has been evaluated in preclinical studies and clinical trials^14,15^, the efficacy of dual LAG-3 and TIGIT blockades is yet unknown. Due to the blocking mechanisms of anti-LAG-3 and anti-TIGIT, it is assumed that simultaneous blockade of LAG-3 and TIGIT would be a novel strategy for cancer immunotherapy. Here, we report the findings of a preclinical study of ZGGS15, a novel bispecific monoclonal antibody (BsAb) that can simultaneously inhibit LAG-3 and TIGIT alone or in combination with an anti-PD-1 antibody, exhibiting exceptional anti-tumor efficacy.

## Materials and methods

### Animals, cell lines and human peripheral blood mononuclear cells (PBMC)

BALB/c-hPD-1/hTIGIT and BALB/c-hPD-1/hLAG-3 mice were provided by GemPharmatech Co., Ltd., and NOG mice were provided by Beijing Vital River Laboratory Animal Technology Co., Ltd. All animal procedures were performed in compliance with Institutional Animal Care and Use Committee (IACUC) guidelines. The protocol was approved by the ethical committee of the First Affiliated Hospital of Zhengzhou University. In vivo anti-tumor responses were evaluated using transplantation of cancer cell lines. Only female mice were used for in vivo experiments. All experiments were performed by relevant named guidelines, regulations, and the ARRIVE guidelines.

On the 10th day after inoculation, when the average tumor volume reached 94.20 mm3, 40 tumor-bearing mice were selected and randomly divided into 5 groups according to tumor volume, with 8 mice in each group. The day of grouping was defined as day 0 (D0). Excess mice were euthanized during the study. The administration of the test agents started on D0. The ZGGS015 dosing dates were: D0, D3, D7, D10, D14, and D17. The Ab1 dosing dates were: D1, D4, D8, D11, D15, and D18. The dosing volume was adjusted according to the weight of the mouse, with a dosing volume of 10 μL/g (ZGGS015) and 5 μL/g (Ab1). Tumor volumes and body weights were measured twice in the first week and adjusted to three times a week in the following weeks. The specific measurement days were D0, D3, D7, D9, D11, D14, D16, D18, D21, and D23. Tumor volume was expressed in mm3 using the formula: TV = 0.5 × a × b2, where a and b were the long and short diameters of the tumors, respectively. To assess the well-being of mice, changes in body weight, behavior, and physical appearance were monitored. All animal experiments were repeated at least three times to ensure the reliability of the results. Animals were sacrificed using a method suggested by the guidelines for euthanasia of rodents using carbon dioxide by the National Institutes of Health (NIH).

An in-house developed CHO-K1 cell expression system was used to create Hu-LAG-3 CHO-K1 cells stably expressing human LAG-3 and Hu-TIGIT CHO-K1 cells stably expressing human TIGIT. Raji (Cat# CCL-86) and SHP-77 (Cat # CRL-2195) cells were purchased from ATCC. All cells were cultured under standard conditions and harvested at the logarithmic growth stage. PBMCs for in vitro assays were provided by StemCell (Cat #70025).

### Construction and expression of ZGGS15

ZGGS15, a novel bispecific antibody (BsAb) targeting LAG-3 and TIGIT, was generated in-house using standard recombinant monoclonal antibody technology, similar as described in the reference^16^. The ZGGS15 protein was expressed in CHO cells and purified through protein A affinity column chromatography. Purity was determined using size-exclusion high-performance liquid chromatography (SEC-HPLC).

### Affinity determination of ZGGS15 to target antigens

Kinetic analyses of ZGGS15 were performed using a GatorPrime biolayer interferometry (BLI) instrument (GatorBio). BLI kinetics assay buffer (1X PBS, 0.1% BSA, 0.02% Tween-20. 0.05% sodium azide) was used to make working protein solutions and was used in all steps of the affinity assay. In LAG-3 affinity assays, purified stocks of ZGGS15 or BM were diluted to 2 µg/mL and loaded to anti-human Fc probes. Following a short baseline step, loaded probes were assayed for binding against three-fold serial dilutions of human LAG-3, ranging in concentration from 100 to 0.41 nM. The LAG-3 association phase took place for 200 s, followed by a 200 s dissociation step. The experiment was performed at 25 degrees Celsius with a shaking speed of 1000 rpm.

For TIGIT affinity determination, probes were loaded with ZGGS15 as described above and assayed against serial dilutions of human TIGIT. TIGIT concentrations ranged from 100 to 1.5 nM, and the association and dissociation steps were assayed for 200 s and 400 s, respectively. In each experiment, a probe containing ZGGS15 assayed against BLI buffer (no analyte) served as a reference subtraction probe. The experiments were performed at 25 °C with a shaking speed of 1000 rpm. The resulting binding curves following reference subtraction were aligned and processed using Savitzky-Goley filtering to generate the final binding curves. Binding curves were fit using a global, 1:1 binding model, and the resulting on- and off-rates were used to determine the binding affinity.

The enzyme-linked immunosorbent assay (ELISA) method was used to measure ZGGS15's binding affinity with target antigens. Briefly, 100 μL/well of human LAG-3 and TIGIT at 0.5 μg/mL in PBS was coated overnight at 4 °C in each well of a 96-well microplate. After washing twice with PBS and blocking with 100 μL of PBS plus 3% (w/v) skim milk for 2 h at room temperature (RT), Serial dilutions of ZGGS15 were added to the plate and incubated at 37 °C for 1 h. After washing, peroxidase-conjugated AffiniPure F(ab')2 fragment goat anti-human IgG Fc-γ fragment-specific antibodies (Jackson Immuno, Cat#109-036-098) were added and incubated at 37 °C for 1 h. Plates were washed six times and TMB solution (KPL, Cat# 51200050) was added and incubated for 15 min (Min) at RT in the dark. Then the reaction was stopped, and the OD450 values were measured. The results were plotted and analyzed using GraphPad Prism software.

### FACS assessment of ZGGS15 binding to human LAG-3 and TIGIT expressing cells

Serial dilutions of ZGGS15 were prepared at fivefold dilutions, resulting in a total of 9 serial dilutions. These dilutions were incubated in plates with Hu-LAG-3 CHO-K1 and Hu-TIGIT CHO-K1 cells for 30 min at 4 °C. After incubation, plates were washed twice with 200 µL of FACS buffer, followed by primary antibody and fluorescent secondary antibody staining procedures. The stained cells were incubated with fixation buffer for 15 min at 4 °C. The plates were washed once with 200 µL of FACS buffer. After removing the supernatant, the cells were resuspended in 200 µL of FACS buffer and analyzed with an Attune NxT Flow Cytometer. FlowJoTM Software (BD Biosciences) was used to determine median fluorescence intensity (MFI) from the AF-647 channel on live cells. Prism software was used for MFI data plotting and determining IC50 values.

### ZGGS15 anti-LAG-3 activity analysis by blocking the interaction of MHC-II and human LAG-3

Serial dilutions of ZGGS15 or anti-LAG-3 benchmark (BM) were prepared at threefold for a total of 12 serial dilutions and added to a 96-well plate with 10 µL huLAG-3-mouse IgG2a (11 µg/mL). After incubating for 30 min at room temperature (RT), 10 µL of the MHC-II-positive Raji cells were added per well to a final concentration of 4 × 10^4^ cells/well and incubated for 40 min at 4 °C. Following incubation, the plates were washed twice with FACS buffer. Fifty µL of PE-labeled goat anti-mouse IgG Fc, diluted in FACS buffer, was added to each well and incubated at 4 °C for 20 min. The plates were washed once with 200 µL of FACS buffer, and cells were resuspended in 30 µL of 1 µg/mL 7-AAD, followed by the addition of 30 µL of a 10% neutral formalin solution to fix the cells before processing the data using a flow cytometer. Prism software was used for MFI data plotting and the determination of IC50 values.

### ZGGS15 Anti-TIGIT activity analysis by blocking the interaction of CHO-K1-hTIGIT and Human PVR

CHO-K1 cells stably expressing human TIGIT were generated in-house. Serial dilutions of ZGGS15 were prepared at threefold for a total of 12 serial dilutions to be tested. CHO-K1-hTIGIT cells were resuspended in FACS buffer to a final cell concentration of 1 × 10^6^ cells/mL and applied to a 96-well plate. Human CD155-mouse IgG2a Fc was added to CHO-K1-hTIGIT cells at a final concentration of 6 µg/mL, followed by 20 µL of ZGGS15 serial dilutions, and incubated with shaking at 4 °C for 30 min. After incubation, plates were washed with FACS buffer, followed by 50 µL of PE-labeled goat anti-mouse IgG Fc, diluted 1:500 in FACS buffer, and incubated at 4 °C for 20 min. The plates were washed with 200 µL of FACS buffer and resuspended in 30 µL of 1 µg/mL 7-AAD, followed by the addition of 30 µL of a 10% neutral formalin solution to fix the cells before processing the data using a flow cytometer. Live cells were distinguished from dead cells using 7-AAD. Median Fluorescence Intensity (MFI) signals were determined from the PE channel (FL-2-H) on live cells. Prism software was used for MFI data plotting and the determination of IC50 values.

### Assessment of ZGGS15 capacity to enhance human T cell responses in vitro

SHP-77 cells were used as targets in the killing assay. PBMCs were cultured alone or in combination with SHP-77 cells at a 5:1 ratio in a round-bottom 96-well plate (1 × 10^5^ PBMCs: 2 × 10^4^ SHP-77 cancer cells). Then, 1 μg/mL of the T cell activator SEB was added alone or in combination with 128 nM of ZGGS15 or blocking antibodies against TIGIT or LAG-3. Alternatively, previously screened PBMCs were cultured with CMV antigens in combination with 100 nM anti-PD-1 mAb alone or in combination with 0.8, 4, 20, or 100 nM ZGGS15. For both activation protocols, PBMCs were cultured for 5 days at 37 °C and 5% CO2. Afterwards, the supernatants were harvested after centrifugation, and cytokines were quantified by ELISA according to the manufacturer’s instructions.

### Potential of ZGGS15 in solution to induce cytokine production from human PBMCs

The induction method of cytokine production from human PBMCs followed a published protocol^17^. IFN-γ, IL-2, IL-6, IL-10, and TNF-α were quantified by sandwich ELISA following the manufacturer's instructions. The AquaMax 4000 (Molecular Devices) plate washer was used for the washing steps of the protocol, and the colorimetric reaction was measured using an EnVision 2105 plate reader (Perkin Elmer). The data from these assays were analyzed using Graphpad Prism version 9.0 (GraphPad Software, Inc., San Diego, CA).

### Plate-bound ZGGS15 to induce cytokine production from human PBMCs

The plates were coated with the testing antibodies overnight at room temperature (RT). The next day, the wells were washed twice with PBS, and 200 µL of PBMC cell suspension (1 × 10^6^/mL) was added per well, followed by incubation at 37 °C and 5% CO_2_. The ELISA assay and data analysis were conducted as described above.

### Antibody-dependent cell-mediated cytotoxicity (ADCC)/complement-dependent cytotoxicity (CDC) assay

100 µL of CHO-K1-hTIGIT cells (1 × 10^6^/mL) were added per well to a V-bottom 96-well plate. After centrifugation, the supernatants were discarded, and 50 µL PBS containing a 1:1000 dilution of the viability dye and a 1/200 dilution of the Fc receptor blocking solution was added per well and incubated for 15 min. Fifty µL FACS buffer containing a 1:200 solution of a commercial anti-TIGIT mAb was added per well and incubated for 20 min, followed by two washes. The cells were then fixed for 30 min at 4 °C in the fixation buffer. After one wash, cells were resuspended in 200 µL FACS buffer per well and acquired with an AttuneTM NxT Flow Cytometer. The data were analyzed using FlowJoTM software (BD Biosciences), following a gating strategy to remove debris and dead cells.

To assess ADCC and CDC, a portion of the target cells were resuspended at 1 × 10^7^ cells per mL in ADCC or CDC assay media, respectively. Fifteen mg/mL mitomycin C was added, and the cells were incubated for 60 min at 37 °C with 5% CO_2_. After two washes, 200 µL of the cell solution (1 × 10^5^) was added per well. PBMCs were added to the ADCC plates, and human complement serum was added to the CDC plates. These plates were incubated at 37 °C and 5% CO_2_ overnight. After the incubation period, the supernatant was collected after centrifugation. Killing was quantified using the CytoTox-Glo assay, following the manufacturer’s instructions. The acquired data were analyzed and plotted using GraphPad Prism software (GraphPad). Then, the luminescence units were normalized to the maximum killing.

### In vitro kinetic analysis to determine the affinity of ZGGS15 to the human neonatal Fc receptor (FcRn)

To determine the affinity of ZGGS15 and BM for human FcRn, binding experiments were performed in a kinetics buffer comprised of 50 mM Sodium Phosphate, 150 mM NaCl, 0.05% Tween-20, 0.01% BSA, at pH 6.0 or pH 7.4, depending on the assay. To assess the interaction of ZGGS15 and BM interacting with the human recombinant FcRn/FCGRT&B2M protein, lyophilized FcRn protein was reconstituted as recommended by the manufacturer to a working concentration of 0.3 µg/mL in assay buffer. Biotinylated FcRn at 0.3 µg/mL was conjugated to streptavidin probes to a loading density of 0.3–0.5 nM. Stocks of ZGGS15 and BM were diluted in kinetics buffer at pH 6.0 (or pH 7.4) to a concentration of 300 nM. Two-fold serial dilutions were made from the 300 nM stock, for a total of seven samples. The seventh sample contained only buffer and no analyte, and served as a sample for background subtraction. The association phase was allowed for 60 s, with a 90-s dissociation step. Probes were regenerated between runs by dipping into 50 mM Tris with 150 mM NaCl, pH 8.0 for 60 s. The interaction assays were performed at 25 °C with a 1000 rpm shaking speed.

To assess how pH influences off-rate, probes conjugated with FcRn and bound to ZGGS15 or BM at pH 6.0 were transferred to wells containing 50 mM sodium phosphate, 150 mM sodium chloride, 0.05% Tween-20, 0.01% BSA, and pH 7.4. In assays where both association and dissociation steps were performed at pH 7.4, the streptavidin probes loaded with FcRn were equilibrated in wells containing the pH 7.4 kinetic buffer for 60 s. Probes were equilibrated to pH 7.4 before being dipped into serial dilutions of ZGGS15 or BM, both in pH 7.4 buffer, for 60 s, followed by a 90-s dissociation phase in pH 7.4 buffer. The interaction assays were performed at 25 °C and a 1000 rpm shaking speed. The data processing method was used as described above.

### In vitro kinetic analysis to determine the affinity of ZGGS15 to human Fc-γ receptors and C1q

To determine the affinity of ZGGS15 and BM for FcγRI, we prepared 4 µg/mL solutions of either antibody by diluting purified protein into BLI kinetics assay buffer. Serial dilutions of FcγRI were made ranging in concentration from 30 to 1 nM. ZGGS15 was loaded to protein A assay probes for 150 s, followed by a short wash step of 60 s. FcγRI binding took place over 150 s, followed by a 200 s dissociation step. The experiment was performed at 25 degrees Celsius with a shaking speed of 1000 rpm. The affinity of ZGGS15 for other members of the Fc-gamma receptor family was determined by BLI as well. As low affinity interactions are expected for IgG4 molecules, high concentration solutions of ZGGS15 and BM were made at 10 µM^1^. Two-fold serial dilutions were made from the 10 µM stocks to provide a range of concentrations for affinity determination. Binding assays with each low affinity FcγR were tested in an identical matter. In a typical experiment, FcγR was prepared at 3 µg/mL in BLI kinetics assay buffer and loaded to BLI anti-His assay probes. Following a 30 s wash step, FcγR was tested for binding to serial dilutions of ZGGS15 or BM for 90 s followed by a 90 s dissociation step.

ZGGS15 and BM binding affinity to human C1q was determined by a similar ELISA method as described above. Briefly, 50 µL per well of ZGGS15 or BM at 10 µg/mL was coated in high-binding 96-well plates at 4 °C overnight. After being washed three times, plates were blocked with 200 µL of ELISA assay buffer for 1 h at RT. Following the blocking step, plates were washed three times, and 50 µL per well of the threefold serially diluted C1q (from 1000 nM to 0.02 nM) was added to the plate and incubated for 1 h at RT. After washing, 50 µL per well of a 1:100 dilution of sheep anti-C1q-HRP secondary detection antibody was added to the plates and incubated for 1 h at RT. After washing, 50 µL/well of Ultra TMB-ELISA substrate solution was added to each well. After two minutes, TMB was followed by adding 50 µL per well of 0.16 M H_2_SO_4_ stop solution. Absorbance at 450 nM was measured, and the results were plotted in Prism.

### In vivo efficacy study of ZGG15 in BALB/c-hPD-1/hLAG-3 mice subcutaneously transplanted with CT26.WT tumor

The mouse colon cancer cells CT26.WT were inoculated subcutaneously into female BALB/c-hPD-1/hLAG-3 mice. When the average tumor volume reached 94.20 mm3, 40 tumor-bearing mice were selected and randomly divided into 5 groups, each with 8 mice (n = 8 per group). High-dose ZSGS15 (5 mg/kg, i.p., BIW × 3 W), low-dose ZSGS15 (2 mg/kg, i.p., BIW × 3 W), nivolumab (1 mg/kg, i.p., BIW × 3 W), and a combination of high-dose ZSGS15 plus nivolumab (5 mg/kg + 1 mg/kg, i.p., BIW × 3 W) were administered. The control group was given the same volume of vehicle (saline) by intravenous injection. Tumor volumes and body weights were measured twice in the first week and adjusted to three times per week in the following weeks. At the study endpoint, photos of tumor-bearing mice and tumors were taken, and the tumor weights were measured.

### In vivo efficacy study of ZGGS15 in BALB/c-hPD-1/hTIGIT mice subcutaneously transplanted with CT26.WT tumor

The mouse colon cancer cells CT26.WT were inoculated subcutaneously into female BALB/c-hPD-1/hTIGIT mice. When the average tumor volume reached 97.41 mm3, 48 tumor-bearing mice were randomly divided into 6 groups (n = 8 per group). Mice in group 1 were treated with vehicle (i.p., BIW × 6 doses). Mice in groups 2 and 3 were treated with 1.5 mg/kg and 5 mg/kg of ZSGS15, respectively (i.p., BIW × 6 doses). Mice in group 4 were treated with 1 mg/kg of nivolumab (i.p., BIW × 6 doses). Mice in groups 5 and 6 were treated with 1.5 mg/kg + 1 mg/kg and 5 mg/kg + 1 mg/kg of ZSGS15 plus nivolumab, respectively (i.p., BIW × 6 doses). Tumor volumes and body weights were measured and analyzed. At the study endpoint, photos of tumor-bearing mice and tumors were taken, and the tumor weights were measured.

### In vivo anti-tumor efficacy for ZGGS15 in PBMC humanized NOG mice bearing the A375 tumor

Ten days before tumor inoculation, each mouse received an intravenous injection of PBMC cells (5 × 10^6^) in 0.2 mL of DPBS. The mice were then inoculated subcutaneously at the right flank with A375 cells (5 × 10^6^) in 0.2 mL of DPBS 1:1 mixed with BD Matrigel for tumor development. Four days post-inoculation, mice were randomly divided into different groups (n = 8 per group) when the average tumor volume reached approximately 106.58 mm3. The test drugs were administered to the mice intravenously once every 5 days. Tumor sizes were measured three times weekly for T/C (%) and TGI (%) value calculation. Body weights were measured three times weekly to assess the mice’s tolerance for drugs.

### Statistical analysis

SPSS and GraphPad Prism 8.0 software (San Diego, CA, USA) were used for statistical analysis, and the results are expressed as mean ± standard deviation. Statistically significant differences between different groups were analyzed using the Student t-test. Kaplan–Meier survival curves were used to compare survival rates between different groups. A p-value < 0.05 indicates a statistically significant difference.

### Ethics approval and consent to participate

Informed consent was obtained for study participation from all donors. Murine studies were conducted after IACUC approved protocol. Blood samples were collected from healthy donors with written consent. Protocol was approved by the First Affiliated Hospital of Zhengzhou University.

## Results

### Characterization of LAG-3/TIGIT bispecific antibody, ZGGS15

**Figure 1:**
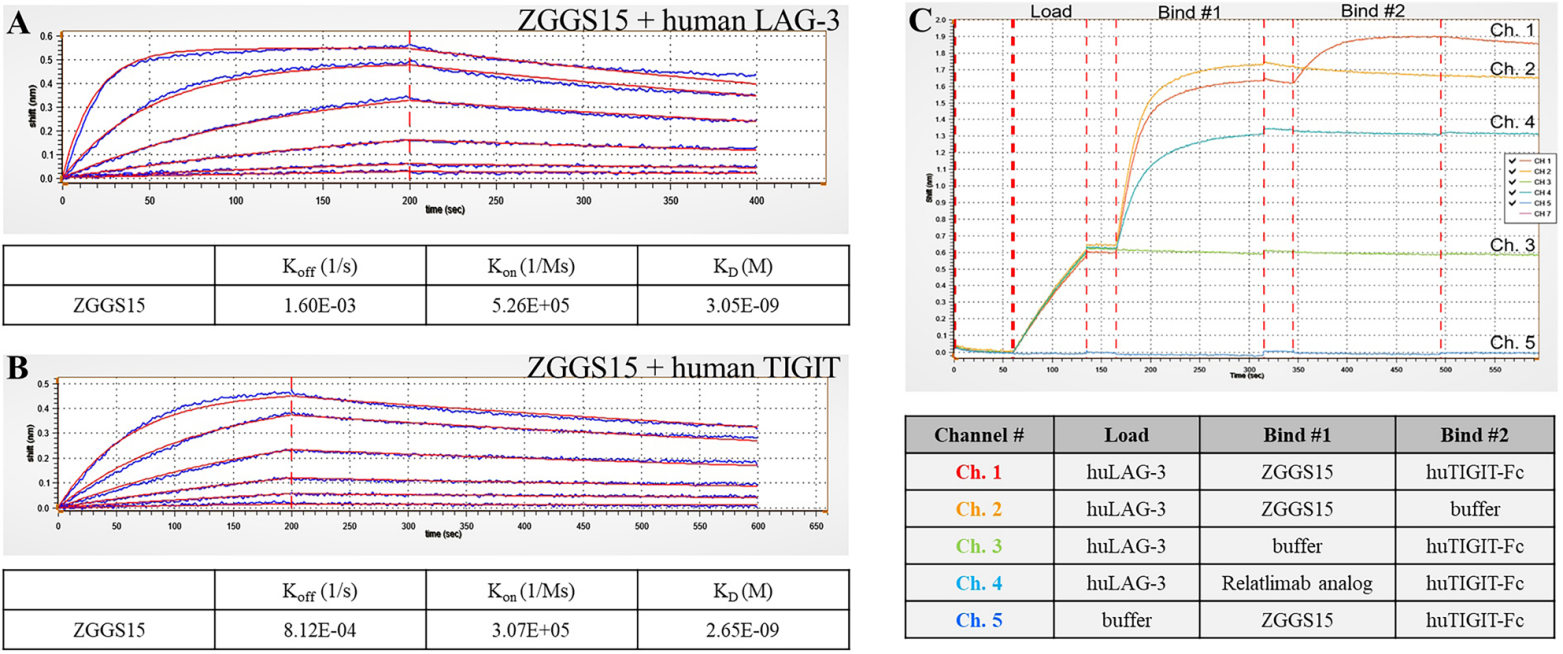
HYPERLINK "sps:id::fig1||locator::gr1||MediaObject::0"BLI assay to determine the affinities of ZGGS15 to human LAG-3 and human TIGIT. (**A**) BLI assay probes containing ZGGS15 were incubated with serial dilutions of human LAG-3 to determine binding affinity of 3.05 nM. (**B**) BLI assay probes containing ZGGS15 were loaded into serial dilutions of human TIGIT to determine a binding affinity of 2.65 nM. (**C**) Simultaneous binding of ZGGS15 to human LAG-3 and human TIGIT. Sensor binding traces are shown above in (**C**), and the binding pattern for each channel is listed below in (**C**). On Channel 1(red trace), the TIGIT-loaded probe associates with ZGGS15 in the “Bind #1” step. In the “Bind #2” step, the binding of human TIGIT to the ZGGS15-LAG-3 complex can be observed.

ZGGS15 is a recombinant humanized bispecific antibody that is composed of a monoclonal antibody against LAG-3 and TIGIT. It can specifically bind LAG-3 and TIGIT. The BLI method was used for the in vitro kinetic analysis to determine the affinity of ZGGS15 for human LAG-3 and human TIGIT. ZGGS15 was found to bind to human LAG-3 with a KD of 3.05 nM (Fig. 1A). Similarly, ZGGS15 binds to human TIGIT with a KD of 2.65 nM (Fig. 1B). These binding affinities indicated that ZGGS15 could bind LAG-3 and TIGIT with high affinity.

As a bispecific antibody, simultaneous binding to a dual target is essential for a combined or synergistic effect. The result demonstrated that ZGGS15 could bind to human LAG-3 and human TIGIT simultaneously (Fig. 1C).

**Figure 2:**
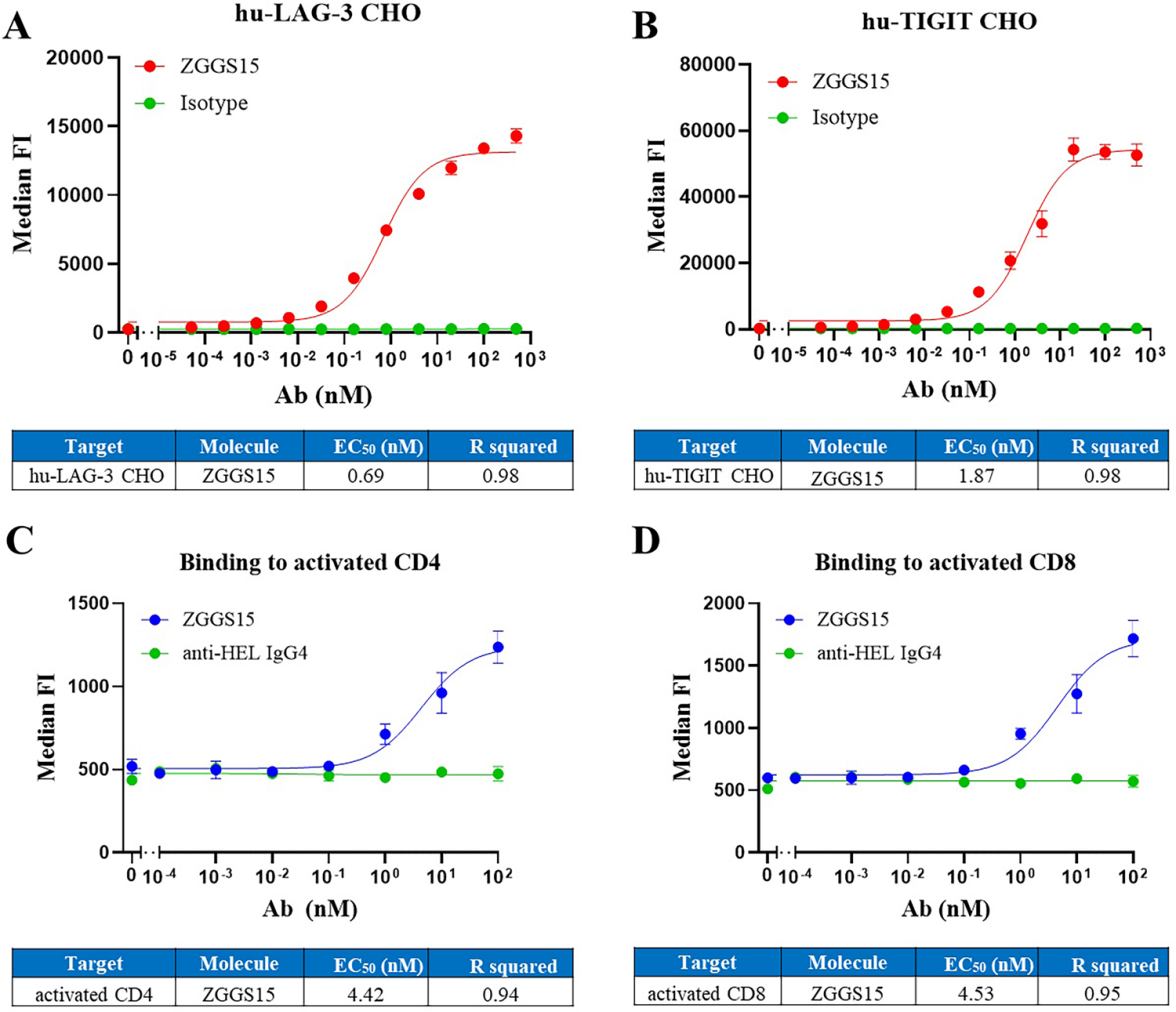
ZGGS15 binds cells expressing LAG-3 and TIGIT with high affinity. CHO cells expressing human LAG-3 (**A**) or human TIGIT (**B**) were stained as described in the Methods section. The median fluorescence intensity (MFI) from the AF-647 channel was calculated using FlowJo, and the means of triplicate wells plus their standard deviation were shown. PBMCs were stained as described in the Methods section. Activated CD4 (**C**) and CD8 (**D**) T cells were identified as CD25 CD4 double positives or CD25 CD8 double positives that were negative for the NK marker CD335. Each dot corresponds to the average of triplicate measures, and the standard deviation is represented in the error bar.

Furthermore, cell-based binding was explored. ZGGS15 bound to human LAG-3 and TIGIT proteins expressed on CHO-K1 cells with a dose-dependent nanomolar EC50 of 0.69 nM (Fig. 2A) and 1.87 nM (Fig. 2B), respectively. Human activated T cells were considered to be an ideal binding target for their clinical relevance, and results indicated that ZGGS15’s on-cell binding to activated CD4^+^ T and CD8^+^ T cells was concentration dependent and had an EC_50_ of 4.42 nM (Fig. 2C) and 4.53 nM (Fig. 2D), respectively, while the isotype antibody did not exhibit any binding activity (anti-HEL IgG4) (Fig. 2C,D).

**Figure 3:**
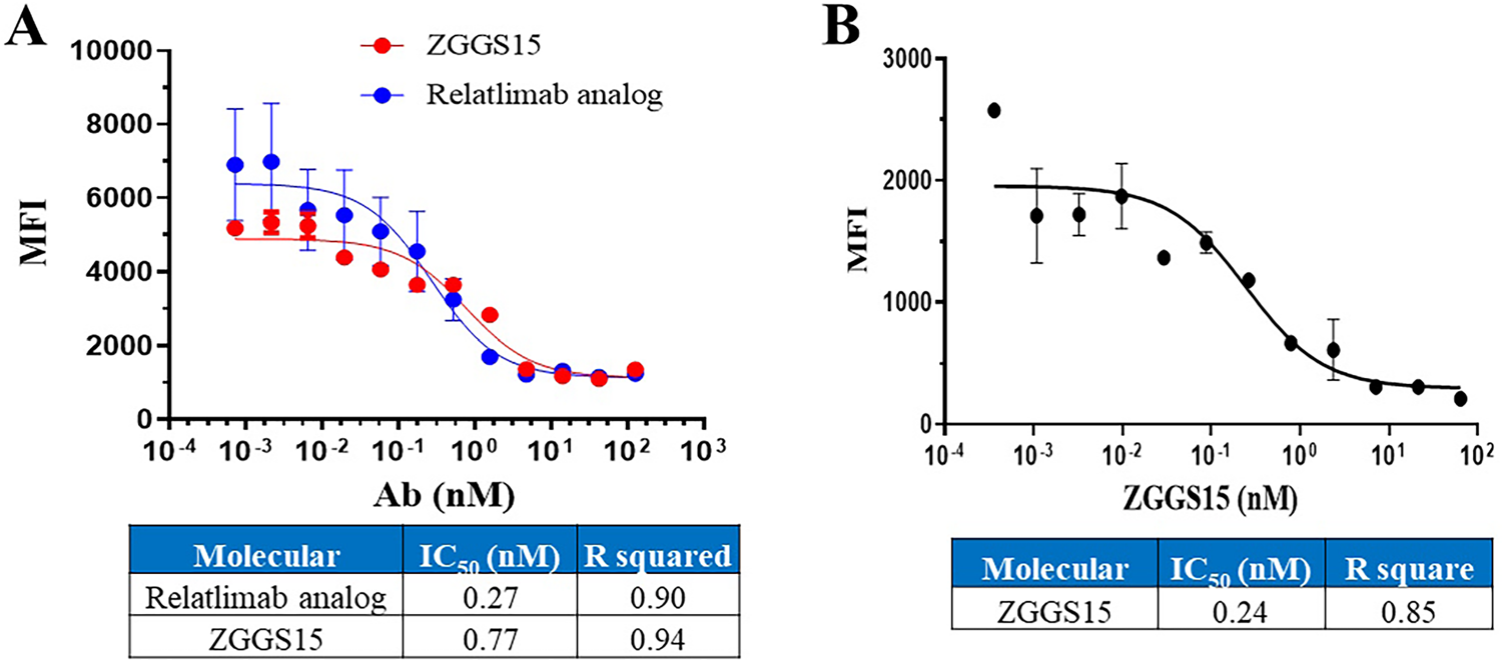
ZGGS15 competitively inhibits the human LAG-3/MHC-II and human TIGIT/CD155 interactions. (**A**) LAG-3 binding to human MHC-II-expressing Raji cells was performed. Plotted is the mean between two replicates wells treated with the same ZGGS15 concentration. The error bar corresponds to the standard deviation between both measurements. (**B**) CD155 binding to human TIGIT-expressing CHO cells was performed. Plotted is the mean between 2 replicates wells treated with the same ZGGS15 concentration. The error bar corresponds to the standard deviation between both measurements.

After binding, a blocking assay between receptor and ligand was conducted. It showed that ZGGS15 competitively inhibited the binding of LAG-3 to MHC-II on the surface of Raji cells (IC_50_ = 0.77 nM), similar to that from the Relatlimab analog (IC_50_ = 0.27 nM), an anti-LAG-3 benchmark (Fig. 3A). Similarly, ZGGS15 also competitively inhibited the binding of CD155 to TIGIT (IC_50_ = 0.24 nM) (Fig. 3B).

**Figure 4:**
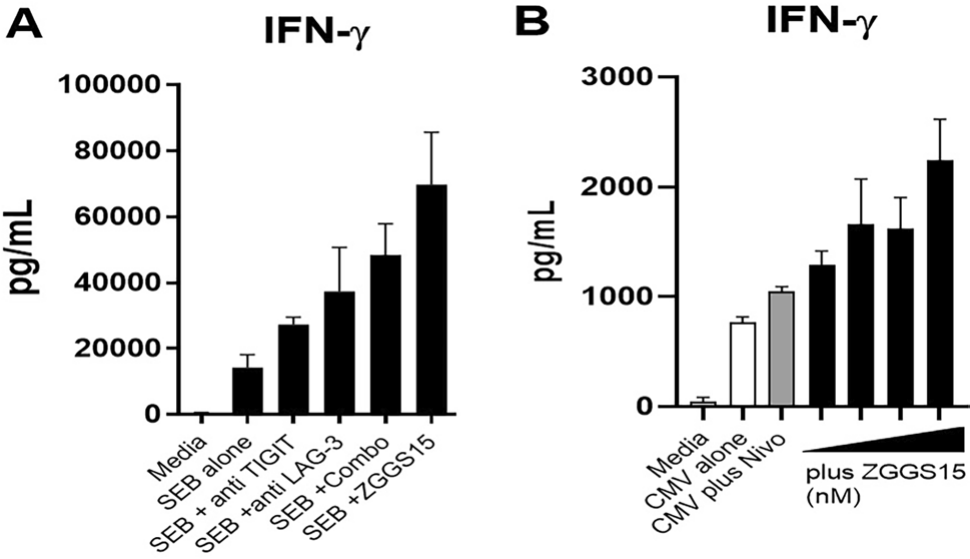
ZGGS15 enhances T cell responses induced by different stimuli. In the presence of the cancer cell line SHP-77, PBMCs were activated with SEB (**A**) or were activated with CMV (**B**) plus the specified antibodies as described in the Methods sections. The concentrations of antibody used in (**A**) experiment were 128 nM for ZGGS15, anti-TIGIT and anti-LAG-3 antibody, respectively. The concentrations of antibody used in experiment (**B**) were 100 nM anti-PD-1 mAb alone or in combination with 0.8, 4, 20, or 100 nM ZGGS15, respectively. Plots show the mean IFN-γ production of triplicate cultures, and the error bar displays the standard deviation between these measurements.

The potency of ZGGS15 to enhance T-cell responses induced by an antigen-independent stimulus (staphylococcal enterotoxin B.SEB) was evaluated. In addition, SHP-77, a TIGIT ligand (CD155, PVR)-positive cancer cell line, was added to the culture (Fig. 4A). Under this condition, ZGGS15 induced better T cell activation (IFN-γ) than a single anti-LAG3 or anti-TIGIT mAb or combination. To evaluate the capacity of ZGGS15 to enhance antigen-specific T cell responses and synergize with other checkpoint inhibitors, Nivolumab (PD-1 blocking antibody) was cultured in PBMCs with CMV antigens plus increasing concentrations of ZGGS15. Nivolumab on its own increased the IFN response towards cytomegalovirus (CMV), and when combined with ZGGS15 can further enhance T cell activation in a dose-dependent manner (Fig. 4B). Therefore, ZGGS15 enhanced T cell responses toward different stimuli both with and without the presence of cancer cells and synergized with nivolumab.

### ZGGS15 displayed a good safety profile and long circulation potential.

ZGGS15 is an IgG4-based molecule; therefore, it should be a poor inducer of Fc-mediated effector functions. In each of the tested assays, ZGGS15 and the Relatlimab analog exhibited similar behavior when interacting with members of the Fc-gamma receptor family. When looking at the affinity of ZGGS15 for the high-affinity receptor FcγRI, it was found that ZGGS15 binds with a high affinity of 2.6 nM. This is very similar to the calculated affinity of 3 nM for the Relatlimab analog and FcγRI (Supplementary Fig. 1). Binding assays with the low-affinity Fc-gamma receptors showed a similar result, where ZGGS15 and Relatlimab analog bound with low affinity in the micromolar range (data not shown). In each assay, ZGGS15 displayed a slightly higher affinity than the Relatlimab analog. This may be due to subtle differences in the glycosylation or fucosylation profiles of the two proteins. For C1q binding, ZGGS15 and Relatlimab analog also demonstrate similar affinities (Supplementary Fig. 2). The low binding affinities measured for Fc-gamma receptors and C1q are in line with the expected values for an IgG4 molecule. Given these results, it is not believed that ZGGS15 could utilize ADCC or CDC mechanisms.

**Figure 5:**
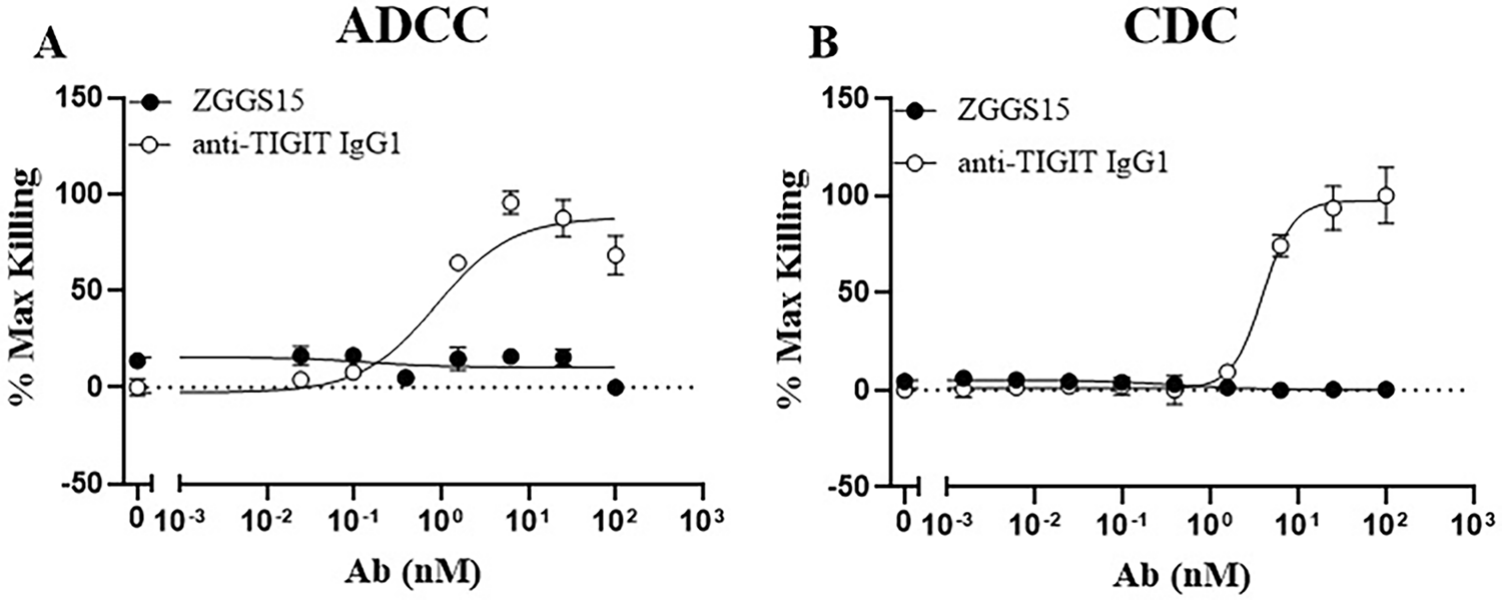
ZGGS15 did not induce killing in ADCC or CDC tests. ADCC (**A**) and CDC (**B**) tests were performed. The data represented by white circles show the killing of TIGIT-expressing cells treated with anti-TIGIT IgG1. The same antibody was used as a positive control on both assays. The luminescence readings were normalized, and the means of triplicate values, along with their standard deviation were presented.

Further, ADCC/CDC assay results showed that ZGGS15 binding to target cells does not induce ADCC or CDC (Fig. 5A,B). The low binding affinities measured for Fcγ receptors and C1q are consistent with ADCC/CDC results and are expected for an IgG4 molecule.

For cancer immunotherapy, cytokine release is another key consideration for safety. Cytokine release syndrome (CRS) is a rapid systemic inflammatory response characterized by the release of pro-inflammatory cytokines (e.g., TNF-α, IL-6, IFN-γ, and IL-2) from immune cells.

Detecting the risk of CRS using PBMCs is complicated by the lower responsiveness of blood-borne immune cells to stimulation compared to immune cells in lymphoid organs. In our study, we used a published method^16^ called “RESTORE” (RESetting T cells to Original REactivity) to increase immune cell responsiveness before exposing immune cells to increasing concentrations of ZGGS15.

**Figure 6:**
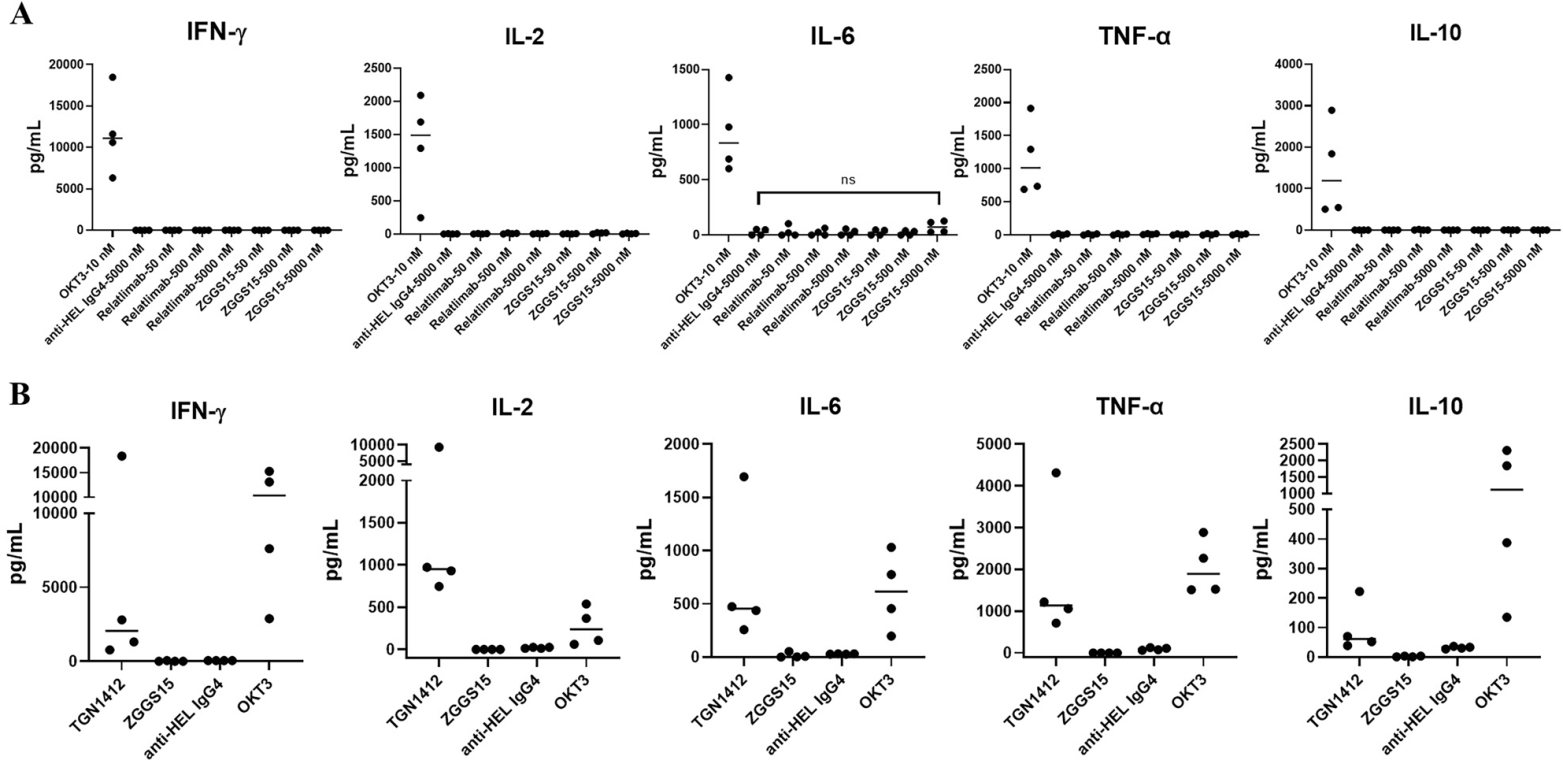
Quantification of ZGGS15-induced cytokine release from human immune cells in a human PBMC-based in vitro model relevant to CRS. (**A**) Human PBMCs were plated at high density and cultured for 48 h. Afterwards, OKT3 (10 nM), anti-irrelevant antigen (anti-HEL IgG4, 5000 nM) or a serial dilution of the antibodies ZGGS15 or Relatlimab analogs were added to the culture. Supernatants were collected 24 h after treatment, and the cytokines were measured by ELISA. Each dot represents one donor, and the line is placed at the median. (**B**) PBMCs from four different donors were added to antibody-coated plates (plate-bound) and cytokine production was quantified after 48 h. E Each dot represents an individual donor, with the line indicating the median. OKT3 and TGN1412 were used as positive controls since both antibodies have induced CRS in a high percentage of patients in their respective clinical trials, while the anti-HEL IgG4-treated group was used to quantify cytokine production in the absence of stimulation (isotype control).

It showed that the supernatants from “RESTORED” PBMCs treated with increasing concentrations of ZGGS15 up to 5000 nM contained similar concentrations of IFN-γ, IL-2, IL-10, and TNF-α as those from Relatlimab analog or irrelevant antibody (anti-HEL)-treated “RESTORED” PBMCs (Fig. 6A). The only cytokine produced by ZGGS15-treated PBMCs that appeared to increase, relative to the irrelevant antibody (anti-HEL IgG4)-treated PBMCs, was IL-6, but only when they were exposed to the highest concentration tested (5000 nM). An unpaired t-test comparison of IL-6 production between irrelevant antibodies (anti-HEL IgG4) and ZGGS15 (5000 nM)-treated PBMCs did not reveal a significant difference. However, “RESTORED” PBMCs treated with an antibody known to activate T cells (OKT3) produced much higher levels of all the cytokines measured than ZGGS15 or Relatlimab, even at a low concentration (10 nM).

In order to more fully assess CRS risks, another CRS evaluation method called plate-bound^18^ stimulation of human PBMCs with monoclonal antibodies bound to the surface of a tissue culture plate, was adopted to explore the CRS potential of ZGGS15. The results revealed that supernatants from PBMCs treated with ZGGS15 (plate-bound) contained concentrations of IFN-γ, IL-2, IL-6, TNF-α, and IL-10 similar to those from isotype antibody-treated PBMCs (anti-HEL IgG4 group). On the contrary, PBMCs that were treated with antibodies known to induce CRS (OKT3, TGN1412^19^) produced obvious levels above the background of all the cytokines measured (Fig. 6B).

**Figure 7:**
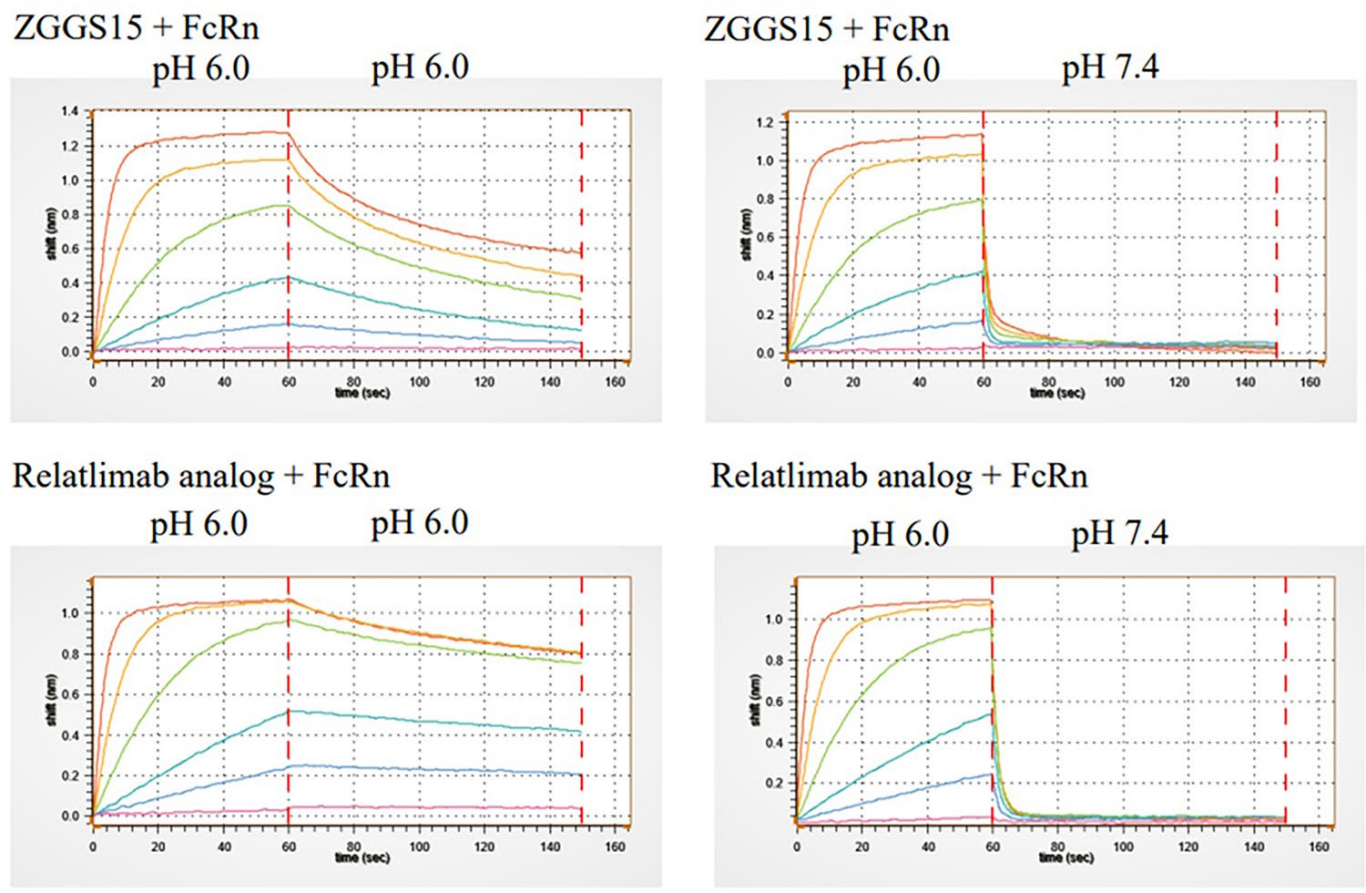
ZGGS15 and Relatlimab analog both display association at pH 6.0 and rapid dissociation at pH 7.4. Figure presents a side-by-side comparison of ZGGS15 and Relatlimab analog, illustrating their pH-dependent interaction with FcRn. The binding curves at pH 7.4 (right images) show rapid initial release compared to dissociation at pH 6.0 (left images).

Serum half-life is another important consideration for Fc-containing antibodies. The ability of Fc-containing molecules to interact with FcRn is an important factor in prolonging serum half-life and molecule efficacy^17,20^. Crucially, the FcRn-IgG interaction operates in a pH-dependent manner, where Fc-containing molecules bind at a lower pH (pH 6.0) and are released at the physiological pH of 7.4^17,20^. This pH-dependent interaction allows for Fc-containing molecules to be recycled back into the serum. To ensure that engineering the ZGGS15 bispecific molecule did not have an impact on FcRn binding, we conducted BLI affinity assays to test ZGGS15 binding to human FcRn. Figure 7 demonstrates that both ZGGS15 and Relatlimab analog bind well to FcRn at pH 6.0, rapidly dissociating at pH 7.4. This pH-dependent interaction is crucial for recirculation.

### Obvious anti-tumor effect of ZGGS15 against the anti-PD-1 weak response model

**Figure 8:**
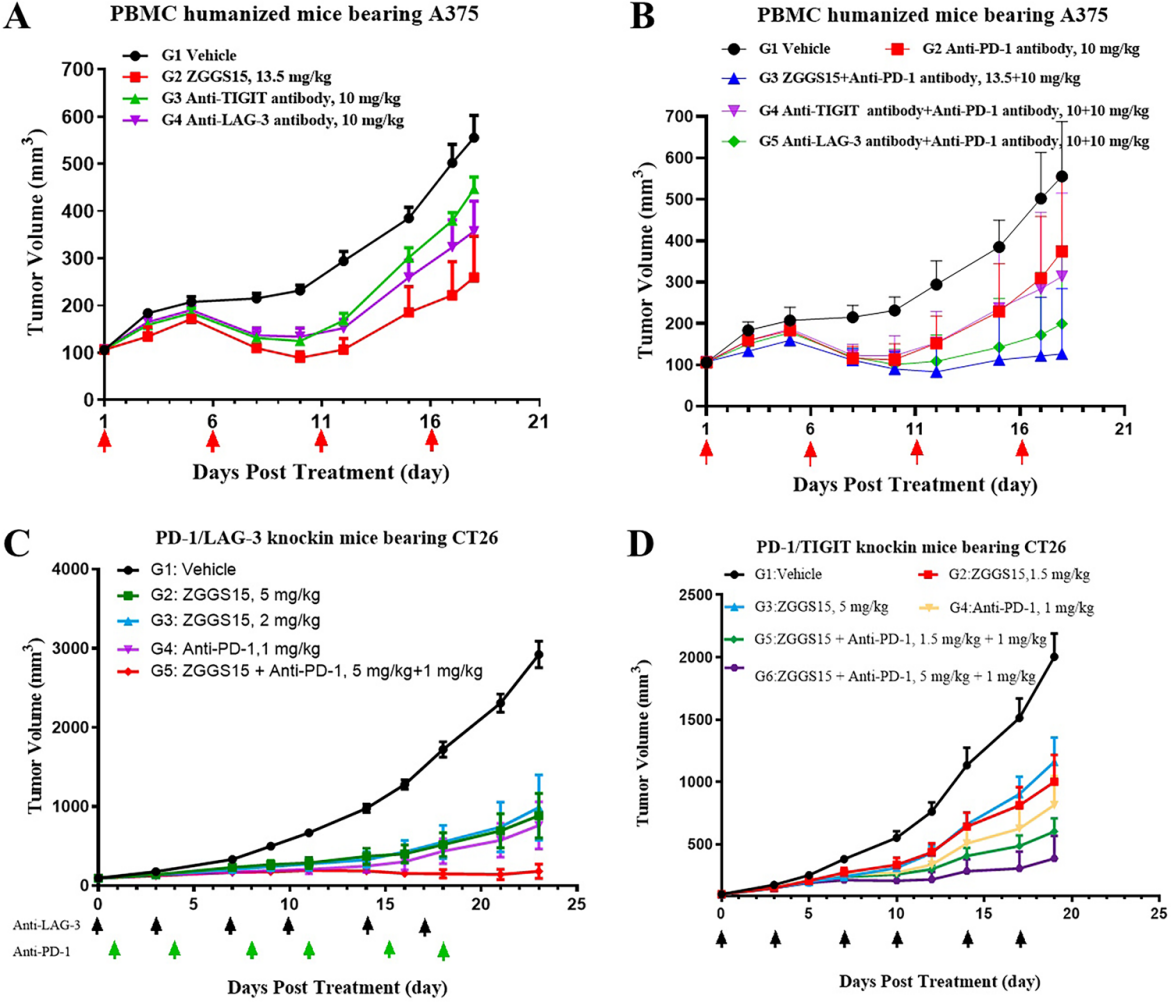
ZGGS15 alone or combined with anti-PD-1 suppressed tumor growth. (**A**, **B**) PBMC-humanized immunodeficient NOG mice were subcutaneously inoculated with melanoma cell A375. Treatment started when the tumor volume reached around 100 mm^3^. Indicated treatments were administered by *i.v.* every five days for a total of four doses. (**C**) PD-1/LAG-3 Balb/c were subcutaneously inoculated with CT26. Treatment started when the tumor volume reached around 100 mm^3^. Indicated treatments were administered by *i.p.* BIW for a total of six doses. (**D**) PD-1/TIGIT Balb/c were subcutaneously inoculated with CT26. Treatment started when the tumor volume reached around 100 mm^3^. Indicated treatments were administered by *i.p.* BIW for a total of six doses.

Finding a next generation immunotherapy agent for anti-PD-1 weak/no response is a big unmet clinical need. This study was to evaluate the anti-tumor efficacy of ZGGS15 against a human PBMC-reconstituted mouse model bearing human melanoma A375, which has a weak response to anti-PD-1. As shown in Fig. 8A, on day 18 post-treatment, the mean tumor volume of the vehicle group reached 555.43 ± 46.91 mm^3^. The ZGGS15 treatment group could obviously inhibit tumor growth, with a mean tumor volume of 259.49 ± 87.28 mm^3^ (TGI = 65.91%). The anti-TIGIT antibody, anti-LAG-3 antibody, and anti-PD-1 antibody treatment groups could only slightly inhibit tumor growth, with mean tumor volumes of 446.66 ± 25.72 mm^3^ (TGI = 24.38%), 357.18 ± 63.73 mm^3^ (TGI = 44.40%), 374.64 ± 66.09 mm^3^ (TGI = 40.23%) respectively. ZGGS15 demonstrated a significant anti-tumor effect in the anti-PD-1 weak response model, outperforming single agents anti-LAG-3 or anti-TIGIT.

### Excellent combined effect of ZGGS15 with an anti-PD-1 antibody

To further explore better efficacy against the anti-PD-1 weak response model, a combined therapy of ZGGS15 plus anti-PD-1 was conducted. As shown in Fig. 8B, in a human PBMC-reconstituted mouse model bearing human melanoma A375, compared to the vehicle group, ZGGS15 plus anti-PD-1 significantly inhibited tumor growth (mean volume 126.13 ± 55.88 mm^3^, TGI = 95.80%, *p* = 0.001), surpassing the efficacy of anti-LAG-3/anti-TIGIT plus anti-PD-1 groups with volumes of 199.22 ± 58.99 mm^3^ (TGI = 79.38%, *p* = 0.006) and 313.89 ± 71.18 (TGI = 54.02, *p* = 0.173).

Another two knock-in mouse models were also adopted to evaluate the combined effect of ZGGS15 plus an anti-PD-1 antibody. First, efficacy for ZGGS15 (only the anti-LAG-3 part is active in this model) plus anti-PD-1 in the BALB/c-hPD-1/hLAG-3 mouse bearing CT26 colon cancer model was performed. As shown in Fig. 8C, at the endpoint (PG-D23), the tumor volume of the vehicle group was 2921.70 ± 168.78 mm^3^, and the tumor volumes of the high-dose ZGGS15, low-dose ZGGS15, anti-PD-1 antibody, and the combination of high-dose ZGGS15 plus anti-PD-1 antibody were 883.57 ± 281.26 mm^3^, 988.33 ± 412.03 mm^3^, 760.49 ± 198.41 mm^3^, and 181.10 ± 91.28 mm^3^, respectively. The tumor volume inhibition rate (TGI) was 69.70%, 65.83%, 74.95%, and 94.03%, respectively. Compared with the vehicle control group, high-dose ZGGS15, anti-PD-1 antibody, and the combination of high-dose ZGGS15 and anti-PD-1 antibody could significantly inhibit tumor growth (*p* < 0.001). Compared with high-dose ZGGS15, the combination of high-dose ZGGS15 and anti-PD-1 antibody could significantly inhibit tumor growth (*p* < 0.05). An excellent combined effect of ZGGS15 with an anti-PD-1 antibody happened in this model. During the experiment, the tumor-bearing mice tolerated well, and no adverse reactions were observed. Second, efficacy for ZGGS15 (only the anti-TIGIT part is active in this model) plus anti-PD-1 in the BALB/c-hPD-1/hTIGIT mouse bearing CT26 colon cancer model was performed. As shown in Fig. 8D, at the endpoint (PG-D19), tumor volume of the vehicle group was 2003.28 ± 186.55 mm^3^; the tumor volumes of the ZGGS15 low-dose (1.5 mg/kg), high-dose (5 mg/kg) groups, anti-PD-1 antibody (1 mg/kg) group, ZGGS15 low-dose plus anti-PD-1 antibody (1.5 mg/kg + 1 mg/kg) and ZGGS15 high-dose plus anti-PD-1 antibody (5 mg/kg + 1 mg/kg) were 1000.18 ± 215.43 mm^3^, 1160.76 ± 194.85 mm^3^, 814.21 ± 232.43 mm^3^, 600.23 ± 107.03 mm^3^ and 384.95 ± 181.10 mm^3^, respectively. The TGI were 51.42%, 41.54%, 58.90%, 70.28% and 81.15%, respectively. Compared to the vehicle group, ZGGS15 low-dose (1.5 mg/kg), high-dose (5 mg/kg), anti-PD-1 antibody (1 mg/kg), ZGGS15 low-dose plus anti-PD-1 antibody (1.5 mg/kg + 1 mg/kg), and ZGGS15 high-dose plus anti-PD-1 antibody (5 mg/kg + 1 mg/kg) could significantly inhibit tumor growth (*p* < 0.001). Compared with the high dose of ZGGS15, the high dose of ZGGS15 plus anti-PD-1 antibody (5 mg/kg + 1 mg/kg) could significantly inhibit tumor growth (*p* < 0.05). This model demonstrated an excellent combined effect of ZGGS15 with an anti-PD-1 antibody. During the treatment, the tumor-bearing mice showed good tolerance to the treatments.

## Discussion

As an immune checkpoint that inhibits activation of T cells and NK cells**,** TIGIT was first identified in 2009^21–23^ and is involved in adaptive tumor immunological surveillance^24^. Anti-TIGIT therapy has been explored as an emerging targetable inhibitory immune checkpoint molecule for cancer immunotherapy^25^. Our data demonstrated that ZGGS15 binds specifically to activated CD4^+^ and CD8^+^ T cell subsets identically with nanomolar affinity. ZGGS15 competitively inhibits human LAG-3 from interacting with human MHC-II. Therefore, we believe that ZGGS15 is expected to block LAG-3 and TIGIT in immune cells binding to their ligands to exert anti-tumor activity.

The ability of Fc-containing molecules to interact with FcRn is an important factor in prolonging serum half-life and molecule efficacy^17,20^. Crucially, the FcRn-IgG interaction operates in a pH-dependent manner, where Fc-containing molecules bind at a lower pH (pH 6.0) and are released at the physiological pH of 7.4^26^. This pH-dependent interaction allows for Fc-containing molecules to be recycled back into the serum. To ensure that engineering the ZGGS15 bispecific molecule did not have an impact on FcRn binding, we conducted BLI affinity assays to test ZGGS15 binding to human FcRn. The steady-state affinities of ZGGS15 and BM are similar at pH 6.0, with no quantifiable binding at pH 7.4. Both molecules demonstrate a strong dissociation from FcRn at pH 7.4. These results suggest ZGGS15 will interact with FcRn in a pH-dependent manner, promoting recirculation of ZGGS15 with a prolonged half-life in plasma.

The selection of IgG subclasses for immune cell targets is very complicated because some targets regulate immune function by transducing inhibitory signals to T cells. Different IgG format tactics should be carefully explored when developing therapeutic antibodies against different immune checkpoints^27^. ZGGS15, an IgG4 BsAb for human LAG-3 and human TIGIT, performs similarly to a native IgG4 in its capacity to interact with effector molecules such as human Fc-γ receptors and the human complement component, C1q. When evaluated by BLI and ELISA, the affinities of ZGGS15 and BM for human Fc-receptors and C1q are comparable. Based on these results, it is expected that adverse events resulting from the effector molecule engagement will be minimal in vivo.

In several animal models, various anti-TIGIT candidate drugs, including OMP-313M32^28^, BGB-A1217 (Ociperlimab)^29^, and 4B1^30^, demonstrated significant anti-tumor efficacy. Anti-TIGIT mAb also enhanced the efficacy of radiotherapy^31^ in high-tumor-burden mice with colon tumors^32,33^^.^ Furthermore, anti-TIGIT therapies have also been evaluated for solid tumors in different clinical studies^14^. Four anti-TIGIT agents, including tiragolumab, vibostolimab, domvanalimab, and ociperlimab^34–36^, have been evaluated in phase II/III clinical trials in PD-L1-positive patients with non-small cell lung cancer (NSCLC)^36^, PD-L1-high locally advanced or metastatic NSCLC^37,38^, locally advanced, unresectable stage III NSCLC, and esophageal squamous cell carcinoma (ESCC), respectively^39^. Furthermore, tiragolumab in conjunction with atezolizumab and bevacizumab also revealed favorable results in hepatocellular carcinoma patients with unresectable, locally progressed, or metastatic disease^40,41^. Three phase III trials, including KeyVibe-006 (NCT05298423), KeyVibe-007 (NCT05226598), and KeyVibe-008 (NCT05224141) were registered to evaluate the efficacy of vibostolimab for NSCLC^17,18,42,43^ or small cell lung cancer^44^. Four phase III clinical trials, including ARC-10 (NCT04736173), PACIFIC-8 (NCT05211895)^45^, STAR-121 (NCT05502237) and STAR-221 (NCT05568095), were registered to assess the effectiveness of domvanalimab and zimberelimab combination therapy for NSCLC and upper gastrointestinal tract cancer. However, no TIGIT-targeted drug has yet been approved by the FDA for cancer immunotherapy.

Meanwhile, anti-LAG-3 therapy has also been explored for cancer immunotherapy in both preclinical studies and clinical trials. Anti-LAG-3 blockade monotherapy didn't demonstrate significant anti-tumor effects, and dual blockade of PD-1 and LAG-3 showed strong synergistic anti-tumor immune responses in preclinical studies^46–48^. Three groups of LAG-3-targeting agents have been evaluated in clinical trials, including anti-LAG-3 blocking mAbs, anti-LAG-3 cell-depleting mAbs, and LAG-3 recombinant fusion proteins^8,15^. Anti-LAG-3-blocking mAbs are the dominant agents in current studies. Seven anti-LAG-3 blocking mAbs are currently being tested, including relatilimab (fully human IgG4 mAb), LAG525 (humanized IgG4), MK-4280 (humanized IgG4), REGN3767 (human IgG4), TSR-033 (humanized IgG4), Sym022 (Fc-inert human mAb), and INCAGN02385 (Fc-engineered IgG1κ)^8^, in addition to anti-PD-1 and LAG-3 BsAbs^8,15^. Furthermore, the anti-LAG-3 cell-depleting mAb GSK2831781^49^ and the LAG-3 recombinant fusion protein IMP321 are also being evaluated in clinical trials^8,15^.

The preliminary results from a phase II/III MAHOGANY trial (NCT04082364) evaluating margetuximab (an Fc-optimized mAb that binds HER2) plus tebotelimab (an IgG4κ bispecific molecule that binds PD-1 and LAG-3 concomitantly) with chemotherapy in first-line unresectable metastatic/locally advanced gastroesophageal junction adenocarcinoma (GEA) revealed potential synergic antitumor activity with good tolerability^50^. A first-in-human study of the anti-LAG-3 mAb favezelimab plus pembrolizumab in previously treated, advanced microsatellite-stable colorectal cancer showed promising antitumor activity, particularly in participants with PD-L1 CPS ≥ 1 tumors^51^. Most importantly, nivolumab/relatlimab was approved by the FDA and the European Medicines Agency following the results of a phase 1/2 trial and a phase 2/3 RELATIVITY-047 trial^52^. The results revealed that relatlimab in combination with nivolumab significantly improved progression-free survival as compared to anti-PD-1 monotherapy in patients with previously untreated metastatic or unresectable melanoma, particularly those with PD-L1 < 1% and/or LAG 3 ≥ 1%., with more clinical trials ongoing^53,54^.

The majority of current studies focus on the anti-TIGIT and anti-LAG-3 target therapy monotherapy or anti-TIGIT plus anti-PD-1 or anti-LAG-3 plus anti-PD-1 combination therapy. We anticipate that the combining anti-TIGIT and anti-LAG-3 dual target therapy will be a novel approach to cancer immunotherapy. Herein, we have demonstrated the in vitro functional binding and blocking activity of ZGG15. BLI results showed ZGGS15 has a high affinity for human LAG-3 and human TIGIT, respectively. ZGGS15 binds specifically to activated CD4^+^ and CD8^+^ T cells with nanomolar affinity. The binding affinity for both activated T cell subsets was practically identical. The binding affinity of ZGGS15 to human LAG-3 and TIGIT proteins expressed by CHO-K1 cell assays shows that ZGGS15 binds target-expressing CHO cells in a dose-dependent manner to human LAG-3 and human TIGIT, respectively. It was reported that elevated fibrinogen-like protein 1 (FGL1), a major LAG-3 functional ligand independent from MHC-II, is associated with a poor prognosis and resistance to anti-PD-1/B7-H1 therapy in cancer patients. The blockade of FGL1 binding to LAG-3 can potentiate anti-tumor T cell responses^55^. Our results showed that ZGGS15 can competitively inhibit the binding of LAG-3 with MHC-II and the binding of TIGIT with CD155, supporting the potential clinical applications of ZGGS15 in cancer patients with high FGL1 expression (e.g., hepatocellular carcinoma)^56^. Combining the aforementioned reports regarding the FGL1 relationship with LAG-3, we speculate that ZG6S15 is expected to work in cancer patients with high FLG1 expression. However, this will need to be verified by more in vitro experiments in the future.

ZGGS15, a BsAb that blocks both LAG-3 and TIGIT, can potently inhibit LAG-3 and TIGIT receptors on immune cells by attaching to their specific binding ligands, MHC-II and PVR (CD155), on tumor cells. Our in vivo efficacy results in mouse models showed that ZGGS15 enhanced the response of T cells to different stimuli in the presence or absence of cancer cells. In addition, ZGGS15 can synergize with the anti-PD-1-blocking antibody nivolumab. The combination of high-dose ZGGS15 and nivolumab was more effective than high-dose ZGGS15 and slightly better than nivolumab in terms of anti-tumor efficacy, and no adverse reactions were observed.

CRS is a rapid systemic inflammatory response characterized by the release of pro-inflammatory cytokines (e.g., TNF-α, IL-6, IFN-γ, IL-2) from immune cells. T cell-engaging therapies can induce CRS, and, most commonly, this response is initiated when the drug reaches the secondary lymphoid organs. Detecting the risk for CRS using PBMCs is complicated by the lower responsiveness to stimulation of blood-borne immune cells relative to immune cells in lymphoid organs. In our study, we used published methods^18,57^ for both the soluble and plat-bound methods to validate the ZGGS15 cytokine induction capability. These findings revealed that ZGGS15 does not induce the release of cytokines, such as IFN-γ, IL-2, IL-6, IL-10, and TNF-α from human immune cells, therefore mitigating any potential adverse effects of CRS.

Collectively, these data support the development of a combination of ZGGS15 plus anti-PD-1 mAb as a promising therapeutic strategy in clinical oncology that has the potential to enhance anti-tumor responses in more cancer types compared with anti-PD-1 mAb monotherapy. Therefore, our data support the potential utility of ZGGS15 in cancer immunotherapy, potentially as the “first-in-class” molecule to enter clinical trials.

## Conclusion

ZGGS15, a novel IgG4 BsAb that targets LAG-3 and TIGIT, has demonstrated exceptional anti-tumor efficacy by inhibiting LAG-3 and TIGIT. ZGGS15 exhibits potent anti-tumor efficacy in transgenic mouse models, either alone or in combination with the PD-1 mAb nivolumab. Furthermore, ZGGS15 does not produce ADCC or CDC, therefore avoiding the non-target binding cross-reaction. Finally, ZGGS15 does not induce the production of cytokines, hence minimizing any potential adverse effects of CRS.

### Supplementary Information

Supplementary Figures.

## Data Availability

The datasets analysed during the current study are available upon request to the corresponding authors.

## Abbreviations

ADCC: Antibody-dependent cell-mediated cytotoxicity
BLI: Biolayer interferometry
BM: Benchmark
BsAb: Bispecific antibody
CDC: Complement-dependent cytotoxicity (CDC)
CMV: Cytomegalovirus
CR: Complete response
CRS: Cytokine release syndrome
CTLA-4: Cytotoxic T-lymphocyte-associated antigen 4
DCs: Dendritic cells
ELISA: Enzyme-linked immunosorbent assay
ESCC: Esophageal squamous cell carcinoma
FcRn: Neonatal Fc receptor
FDA: Food and drug administration
FGL1: Fibrinogen-like protein 1
IACUC: Institutional animal care and use committee
Ig: Immunoglobulin
LAG-3: Lymphocyte activating gene 3
mAb: Monoclonal antibody
MFI: Median fluorescence intensity
Min: Minutes
NSCLC: Non-small cell lung cancer
PBMC: Peripheral blood mononuclear cells
PD-1/L1: Programmed death receptor-1/programmed death protein ligand-1
PVR: Poliovirus receptor
PVRL2: Poliovirus receptor-related 2
RT: Room temperature
SEB: Staphylococcal enterotoxin B
SE-HPLC: Size-exclusion high-performance liquid chromatography
TIGIT: T cell Ig and ITIM domain
TILs: Tumor infiltrating lymphocytes
