# Supplementary material for: A novel anti-LAG-3/TIGIT bispecific antibody exhibits potent anti-tumor efficacy in mouse models as monotherapy or in combination with PD-1 antibody
Source: Sci Rep. 2024 May 9;14:10661. doi: 10.1038/s41598-024-61477-6 (PMC11082181; doi:10.1038/s41598-024-61477-6)
Supplement: Supplementary file 1 — Supplementary Figures. [file 41598_2024_61477_MOESM1_ESM.docx]

Supplementary figures


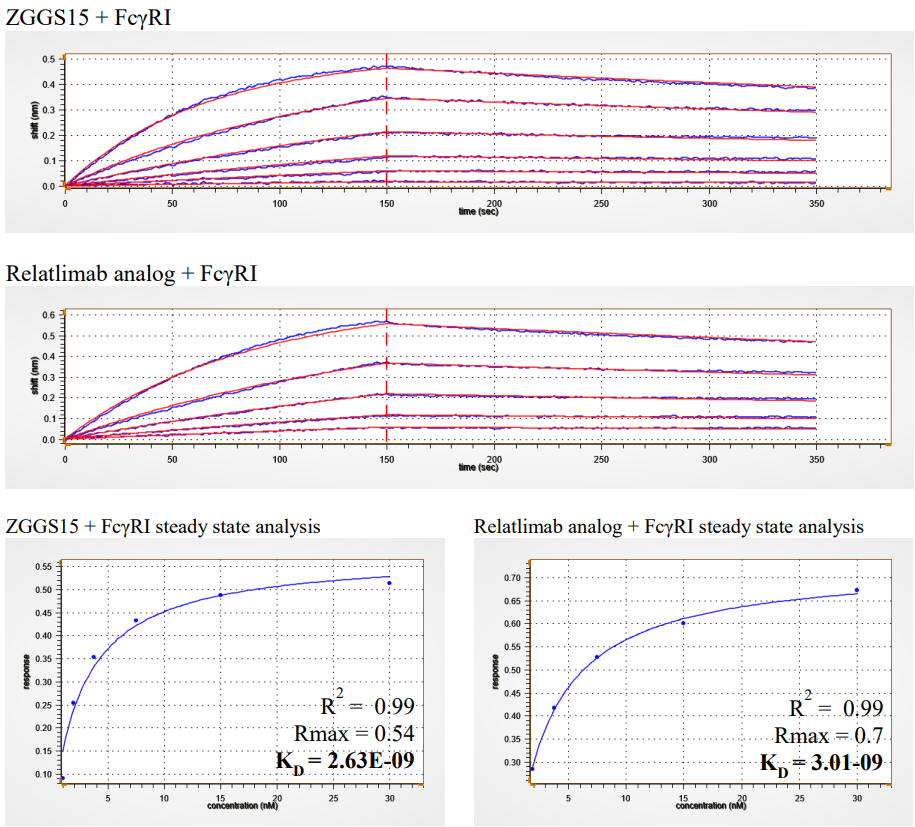


**Supplementary Fig. 1. ZGGS15 and Relatlimab analogs bind to human FcγRI with high affinity.**

ZGGS15 and Relatlimab analogs were loaded onto Protein A probes and assayed for binding against serial dilutions of FcγRI. The binding curves of ZGGS15 and Relatlimab analog are shown in blue (top), with calculated curve fitting shown in red. Steady-state plots of the interaction and calculated affinity constants are presented at the bottom.


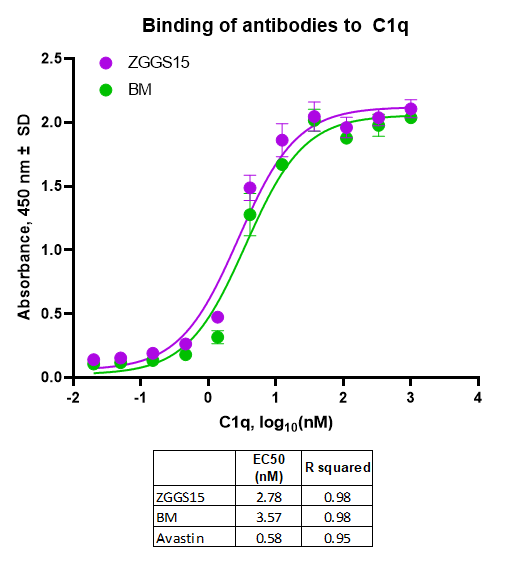


**Supplementary Fig. 2. ZGGS15 and Benchmark (BM, Relatlimab) display similar affinity for C1q**. ZGGS15 and Benchmark were plated at 10 µg/mL and assayed against serial dilutions of human C1q. The resulting binding curves are displayed above, with ZGGS15 in purple and Benchmark in green. The EC_50_ values determined from this assay are presented in the table.
